# Safety of soy-derived phosphatidic acid supplementation in healthy young males

**DOI:** 10.1186/1550-2783-10-S1-P6

**Published:** 2013-12-06

**Authors:** Joshua E Dudeck, Jordan M Joy, Ryan P Lowery, Eduardo O De Souza, Ralf Jäger, Sean A McCleary, Stephanie M C Wilson, Martin Purpura, Jacob M Wilson

**Affiliations:** 1 Department of Health Sciences and Human Performance, The University of Tampa, Tampa, FL, USA; 2 Laboratory of Neuromuscular Adaptations to Strength Training, School of Physical Education and Sport, University of São Paulo, São Paulo, Brazil (EDS; 3 Increnovo LLC, 2138 E Lafayette Pl, Milwaukee, WI, USA; 4 Department of Nutrition, IMG Performance Institute, IMG Academy, Bradenton, FL, USA

## Background

The mammalian target of rapamycin (mTOR) has been shown to regulate rates of muscle protein synthesis, and one novel nutritional activator of mTOR is the phospholipid Phosphatidic Acid (PA). We have recently found that PA supplementation over 8 weeks of resistance training augmented responses in skeletal muscle hypertrophy and strength. However, we are unaware of research investigating the safety of PA in human subjects. Therefore the purpose of this study was to investigate the effects of 8 weeks of 750 mg per day of PA supplementation on safety parameters in healthy college aged males.

## Methods

Twenty-eight healthy, college aged male subjects (21 ± 3 years of age, bodyweight of 76 ± 9 kg, and height of 176 cm ± 9 cm) participated in this study. Subjects were equally divided into experimental and control conditions. The experimental condition (EXP) received 750 mg of soy-derived PA (Mediator™, Chemi Nutra, White Bear Lake, MN), while the control condition (CON) received a visually identical placebo (rice flour). Measures of cardiovascular, kidney, and liver function were analyzed with a full CMP and CBC prior to and 8 weeks following supplementation. This analysis included: total, high density, and low density lipoproteins, blood glucose, blood urea nitrogen, creatinine, eGFR, Na, K, Cl, CO2, Ca, protein, albumin, globulin, albumin:globulin ratio, total bilirubin, alkaline phosphatase, aspartate aminotransferase, and alanine aminotransferase. In addition a sample of urine was submitted for analysis of urine specific gravity and pH. A 2x2 repeated measures ANOVA was used to determine group, time, and group x time interactions. A Tukey post-hoc was used to locate differences.

## Results

There were no differences at baseline in blood chemistry and hematology between the CON and EXP supplemented groups. Additionally no differences were observed in urinalysis values between the groups. Moreover no group, or group X time effects were found following 8 weeks of supplementation.

## Conclusions

Soy-derived PA is a safe nutritional supplement for healthy college aged subjects if taken up to a dosage of 750 mg over an eight week period.

